# Turning the tide (and the probe)—sonographic liver measurement in the right lateral anterior axillary line is a reliable alternative to the standard frontal approach in term and preterm infants

**DOI:** 10.1007/s40477-025-01047-2

**Published:** 2025-07-05

**Authors:** Alexandros Rahn, Thomas Müller, Svea Kleiner, Doris Franke

**Affiliations:** 1 Department of Pediatric Pulmonology, Allergology and Neonatology, Hannover Medical School, Hannover, Germany; 2 Department of Pediatric Cardiology and Pediatric Intensive Care, Hannover Medical School, Hannover, Germany; 3 Department of Pediatric Kidney, Liver and Metabolic Diseases, Hannover Medical School, Hannover, Germany

**Keywords:** Neonatal ultrasound, Liver length measurement, Anterior axillary line, Preterm infants

## Abstract

**Purpose:**

In term and preterm infants, liver size is important for assessing growth and detecting conditions such as hepatomegaly, infections, or cardiac failure. The German Society for Ultrasound in Medicine (DEGUM) recommends measuring craniocaudal liver length in two frontal planes: the midsternal line (MSL) and the right anterior axillary line (AAL). This study evaluated whether liver length measurements in the right AAL from a lateral view yield results comparable to the standard frontal view, as the lateral approach may be technically more accessible and less stressful for neonates.

**Material and Methods:**

In this prospective single-center study conducted in a level III neonatal intensive care unit, 62 term and preterm infants underwent 107 liver measurements between July and December 2024. Each measurement was performed in both the frontal and the alternative lateral AAL plane. Statistical analysis included Wilcoxon matched-pairs signed rank test, Pearson Correlation Coefficient, and Bland–Altman analysis.

**Results:**

The median liver length was 4.7 cm in both methods (interquartile range 1.5 cm frontal, 1.4 cm lateral). The correlation between approaches was excellent (*r* = 0.9971), and Bland–Altman analysis showed a median difference of 0 cm, with 99.1% of measurements within the limits of agreement (− 0.3 cm to 0.2 cm).

**Conclusion:**

Sonographic liver measurement in the lateral AAL yields results equivalent to the standard frontal method and may offer a practical alternative when standard access is limited. Supporting minimal handling of newborns, the approach is particularly relevant in neonatal intensive care and provides a methodological basis for further evaluation of the lateral view.

**Supplementary Information:**

The online version contains supplementary material available at 10.1007/s40477-025-01047-2.

## Introduction

Liver ultrasound with liver length measurements is an important component of diagnostic imaging in preterm and term infants, since hepatomegaly is associated with diseases such as infections (e.g., sepsis, CMV, HSV, toxoplasmosis, syphilis, HIV) [1–6], liver lesions (e.g., abscesses, tumours, vascular malformations) [7, 8], metabolic diseases (e.g., glycogen storage diseases, galactosaemia, cystic fibrosis) [9] or cardiac failure [10].

In neonatal intensive care, liver length assessment can help detect early signs of systemic illness, guide further diagnostic steps (e.g., laboratory testing, echocardiography), and monitor disease progression. This is particularly useful in preterm infants, who often present with nonspecific clinical signs such as temperature instability, feeding difficulties, or lethargy. In these cases, liver ultrasound offers a valuable, non-invasive tool to help identify underlying systemic conditions and guide further diagnostic and therapeutic decisions. Moreover, serial liver length measurements can support longitudinal monitoring of organ development, making the method useful not only in the context of disease, but also for routine follow-up and growth assessment in neonatal care.

The German Society for Ultrasound in Medicine (DEGUM) recommends measuring craniocaudal liver length in two frontal planes: the midsternal line (MSL), using the abdominal aorta as a reference, and the right anterior axillary line (AAL), using the right kidney as a landmark [11]. Reference values for liver length in preterm infants are scarce, and available studies from other regions often employ different anatomical landmarks and measurement planes, limiting direct applicability and comparability [12].

However, in neonatal patients—especially in intensive care settings—frontal access to the liver may be limited or impossible due to adhesive electrodes, stoma appliances, or postoperative abdominal coverings. These cannot always be removed or repositioned, making standard liver measurement unfeasible. Additionally, repeated manipulation of adhesives can cause discomfort or skin damage in preterm and newborn infants.

Due to the close anatomical relationship and easier access, a lateral view offers a potentially less stressful and more accessible alternative. It avoids interference with medical attachments and allows simultaneous visualization of the right kidney, which may be helpful in clinical assessment. Despite these potential clinical advantages, no studies have systematically compared liver length measurements in the lateral versus frontal AAL view in neonates. The aim of this study was therefore to evaluate the agreement between both approaches in term and preterm infants, to determine whether the lateral measurement can serve as a methodologically consistent and clinically promising alternative to the standard method.

## Material and methods

This prospective study included term and preterm infants from a level III neonatal intensive care unit (NICU) and the maternity ward of a tertiary care center between July and December 2024. With approximately 2.500–3.000 births per year and over 500 newborns admitted annually, including around 100 very low birth weight infants (< 1.5 kg) and neonates with various congenital anomalies, the study benefits from a highly heterogeneous patient cohort. Infants were included based on clinical availability during routine care, provided they could be examined by the designated investigator. Children with conditions that could affect liver length (e.g., situs inversus abdominalis, liver or abdominal tumours, metabolic diseases associated with hepatopathy) were excluded. The Ethics Committee did not raise any concerns regarding the project. All legal guardians received written information and consented to the study.

### Ultrasound device and examination procedure

**Fig. 1:**
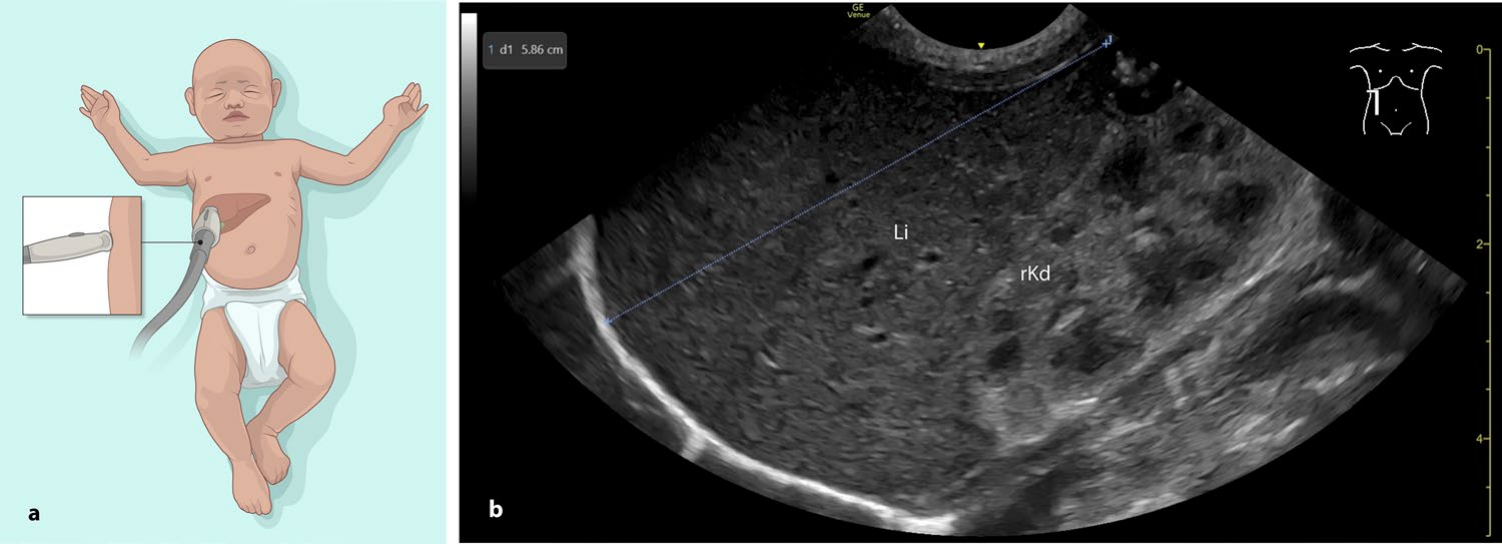
Measuring the liver length in the right AAL from the front. **a** infant in supine position with a convex probe positioned frontal in the right AAL (anterior–posterior plane). **b** sonographic craniocaudal liver length measurement. AAL: anterior axillary line, Li: liver, rKd: right kidney

**Fig. 2:**
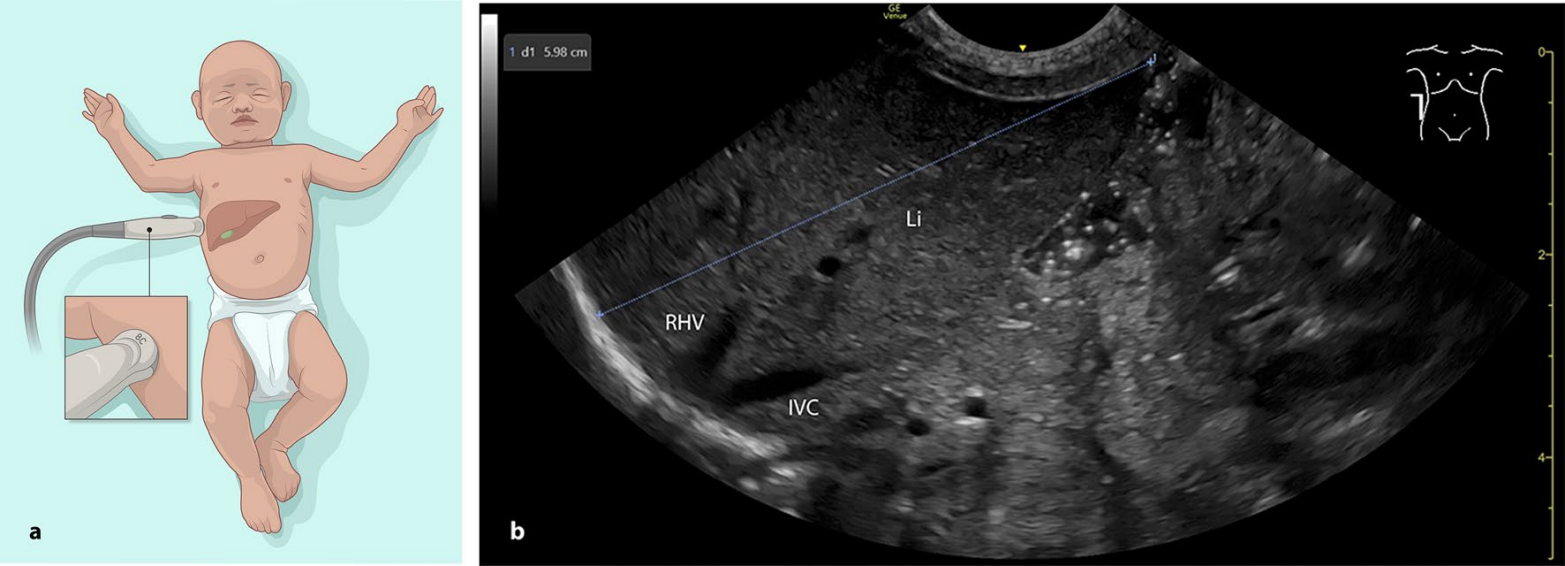
Measuring the liver length in the right AAL in the lateral plane. **a** infant in supine position with a convex probe positioned in the right AAL from the flank (lateral-medial plane). **b** sonographic craniocaudal liver length measurement. AAL: anterior axillary line, IVC: inferior vena cava, Li: liver, RHV: right hepatic vein

Craniocaudal liver length measurements were obtained using a GE Venue Go R4^™^ ultrasound scanner (GE HealthCare Technologies, Chicago, Illinois, USA) and a convex probe (8C). All examinations were performed by a single experienced examiner, with more than 5.5 years of pediatric ultrasound experience and DEGUM level 1 certification, tensuring consistency across measurements. Infants were examined in a supine position and in accordance with the nursing care. For the frontal AAL measurement, the probe was placed sagittal on the right upper abdomen with the ultrasound beam directed anterior to posterior (Fig. 1a + b). For the lateral AAL measurement, the probe was placed sagittally on the right flank (parallel to the mattress), directing the ultrasound beam from lateral to medial (Fig. 2a + b). Measurements were recorded in centimeters and rounded to one decimal place (1—4 = rounded down, 5—9 = rounded up). In a separate group of 17 neonates, liver length was independently measured by a second experienced examiner (DEGUM level 1 certification, > 9 years of pediatric ultrasound experience) to assess interobserver agreement. Both examiners performed liver length measurements in frontal and lateral AAL views within a 24-h interval. The setup also allowed for within-examiner comparison of frontal and lateral measurements to assess consistency across scanning planes.

### Statistical analysis

All statistical analyses were performed using GraphPad Prism 10.4.1 (GraphPad Software, Boston, MA, USA). Data were tested for normal distribution using the Shapiro–Wilk test. Normally distributed variables are reported as mean ± standard deviation; non-normally distributed data are presented as median and interquartile range (IQR).

### Main analysis

Due to non-normal distribution, agreement between frontal and lateral measurements in the primary cohort (*n* = 107) was assessed using the Wilcoxon matched-pairs signed rank test (Wilcoxon test), the Pearson Correlation Coefficient (PCC), and Bland–Altman analysis. For the Wilcoxon test and PCC, only one measurement per patient was included to ensure statistical independence (mean value in case of repeated examinations). Bland–Altman analysis was performed using all paired measurements, as this method does not require independent samples. Since the differences were not normally distributed, limits of agreement (LoA) were defined as the 2.5th and 97.5th percentiles, which are robust to asymmetry and outliers [13].

### Interobserver and within-examiner consistency

For the interobserver substudy (*n* = 17), PCC, paired t-tests, and Intraclass Correlation Coefficients (ICC) were used to assess agreement between two examiners for both frontal and lateral measurements. In addition, a within-observer method comparison (frontal vs. lateral measurements by each examiner) was performed using PCC and paired t-tests to evaluate consistency across scanning planes when applied by the same examiner. Agreement was further visualized using Bland–Altman plots. LoA were reported as mean ± 1.96 SD or based on the 2.5th and 97.5th percentiles, depending on whether the distribution of differences was normal or non-normal.

## Results

### Study population

A total of 107 ultrasound examinations were performed in 62 infants, 27 of whom were examined more than once. Of all participants, 42% were female and 58% male. Eight infants (13%) were born at term, while 54 (87%) were preterm. The median gestational age was 28 ^4^/_7_ weeks (IQR 26 ^2^/_7_–31 ^6^/_7_), the median birth weight 1.05 kg (IQR 0.74–1.55) and the median birth length 36 cm (IQR 32.6–41.5). The first examination was conducted at a median age of 24 days of life (IQR 5–71).

In all 107 examinations, liver length was measured sonographically in both the frontal and lateral AAL planes. The median frontal length was 4.7 cm (IQR 4.1–5.6), the median lateral length was also 4.7 cm (IQR 4.2–5.6). The median difference between the two measurements (frontal AAL minus lateral AAL) was 0 cm (IQR − 0.1 to 0). (Table 1).

**Table 1:** Demographic and clinical characteristics of the study population

Characteristics	Value^*^
Number of infants	62
Number of examinations	107
Infants with multiple examinations Time between first and last examination (days)	27 (44%) 31.81 ± 16.17 Range: 6–72
Term or preterm	Term: 8 (13%) Preterm: 54 (87%)
Sex	Female: 26 (42%) Male: 36 (58%)
Gestational age (p.m., weeks)	28 ^4^/_7_ (26 ^2^/_7_–31 ^6^/_7_) Range: 23 ^4^/_7_–41 ^2^/_7_
Birth weight (kg)	1.05 (0.74–1.55) Range: 0.45–5.4
Birth length (cm)	36 (32.6–41.5) Range: 29–56
Age at first examination (days)	24 (5–71) Range: 1–315
Weight at first examination (kg)	1.99 (1.1–3.7) Range: 0.65–9.27
Length at first examination (cm)	43.5 (36.1–53) Range: 32–72
Liver length in frontal AAL (cm), *n* = 107	4,7 (4.1–5.6) Range: 2.8–8.8
Liver length in lateral AAL (cm), *n* = 107	4.7 (4.2–5.6) Range: 3–9.3
Difference (frontal AAL—lateral AAL, cm), *n* = 107	0 (− 0.1 to 0) Range: − 0.5 to 0.2

*Non-normally distributed data are shown as median (IQR), whereas normally distributed data are presented as mean ± SD. AAL: anterior axillary line

**Fig. 3:**
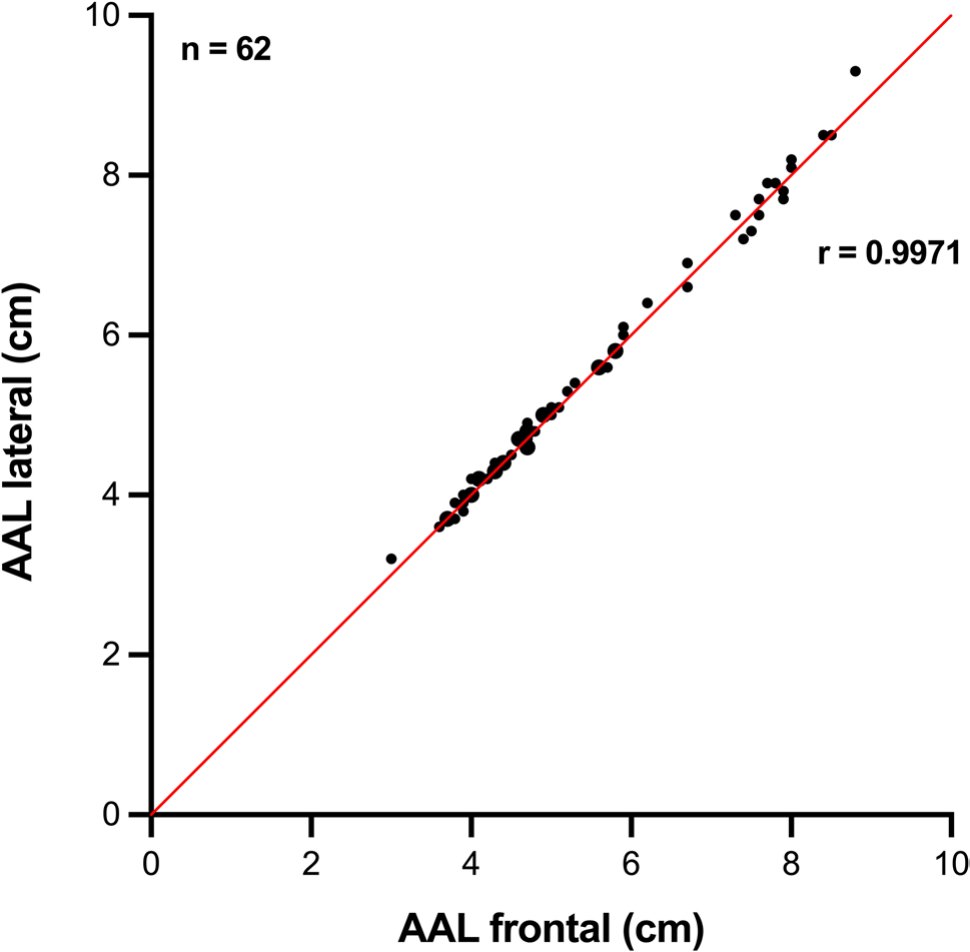
Pearson Correlation between the frontal and lateral liver measurements based on 62 independent data points (aggregated mean values per child): The high correlation coefficient (*r* = 0.9971) shows an almost perfect positive linear correlation between the two measurement methods. Larger points indicate overlapping data from identical measurements. AAL: anterior axillary line

The Wilcoxon test showed a statistically significant deviation from zero (*p* = 0.0052), although the absolute differences were minimal (mean difference − 0.038 cm; frontal AAL minus lateral AAL). PCC revealed an almost perfect positive linear relationship between both methods (*r* = 0.9971, *p* < 0.0001) (Fig. 3).

**Fig. 4:**
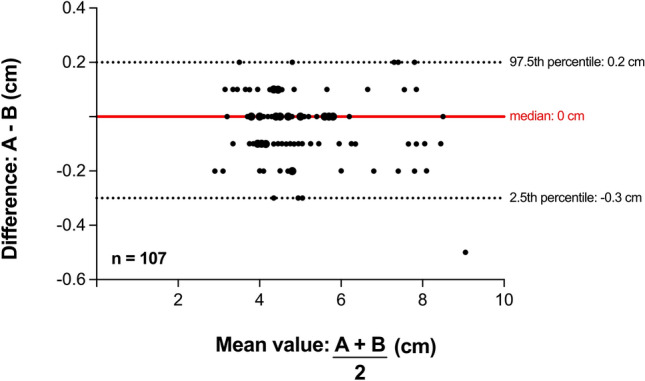
Bland–Altman plot of the differences between the two measurement methods (frontal AAL—lateral AAL, *n* = 107): Each point represents a pair of measurements, with the x-coordinate representing the mean value of the frontal (A) and lateral (B) measurements (cm) and the y-coordinate showing the difference between the frontal and lateral measurements (cm). The middle line shows the median difference (0 cm), the dashed lines indicate the limits of agreement, based on the 2.5th and 97.5th percentiles (− 0.3 cm and 0.2 cm). 106/107 (99.1%) of the measurements fall within this range. Larger points indicate overlapping data from identical measurements. AAL: anterior axillary line

The Bland–Altman analysis demonstrated a median difference of 0.0 cm. The LoA ranged from − 0.3 cm to 0.2 cm, based on the 2.5th and 97.5th percentiles. Overall, 106 of the 107 measurements (99.1%) fell within this range, indicating a high degree of agreement between the two measurement methods (Fig. 4).

**Fig. 5:**
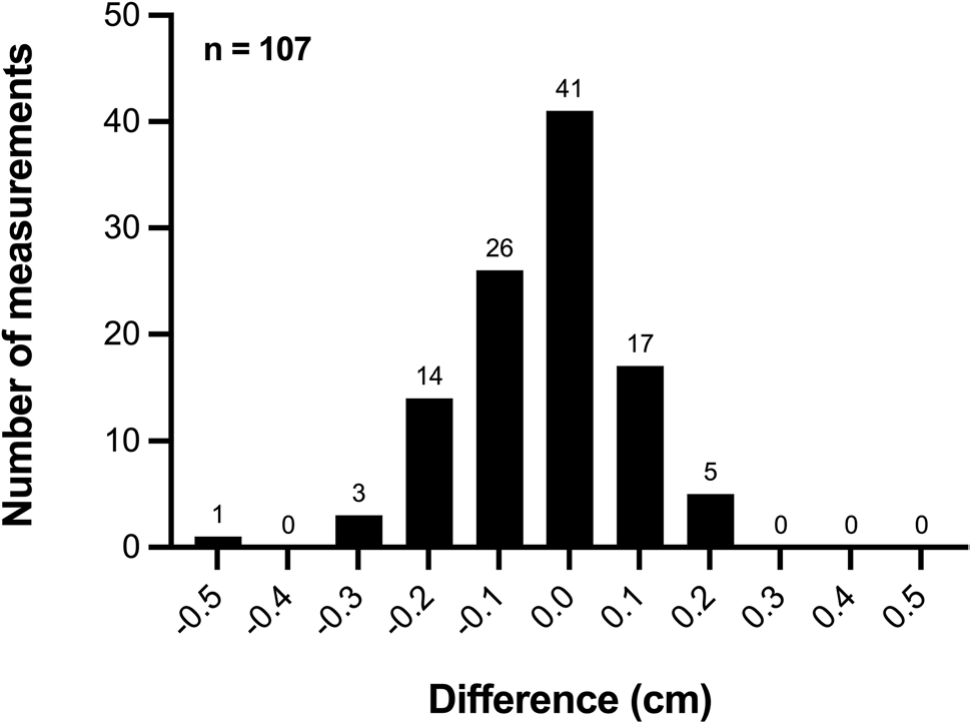
Frequency distribution of the differences (frontal AAL—lateral AAL, *n* = 107): The x-axis shows the differences (cm), while the y-axis shows the frequency of the respective differences. Positive values mean that the frontal method provides higher values, negative values mean that the lateral method provides higher values. AAL: anterior axillary line

In 78.5% of the measurements the difference was ≤ 0.1 cm, and in 96.3% of the examinations the difference was ≤ 0.2 cm (Fig. 5).

### Interobserver and within-examiner consistency

To assess reproducibility, an interobserver substudy was subsequently conducted in a separate group of 17 neonates. Demographic characteristics of this subgroup are presented in Supplementary Table S1. Each examiner independently performed both frontal and lateral AAL measurements. PCC and ICC were > 0.90 for both planes. Paired *t*-tests showed no significant differences between examiners (Supplementary Table S2). Frontal and lateral measurements were also compared separately within each examiner. PCC exceeded 0.90, and no significant differences were observed (Supplementary Table S3). Bland–Altman analysis was performed for all four comparisons: Examiner A (frontal vs. lateral), Examiner B (frontal vs. lateral), frontal measurements between examiners, and lateral measurements between examiners. In each analysis, at least 15 out of 17 measurements (≥ 88%) fell within the calculated LoA. Corresponding plots are presented in Supplementary Figure S1.

## Discussion

This study is the first to systematically investigate whether liver ultrasound measurement in the lateral AAL with a lateral-medial plane provides comparable results to the established frontal measurement in an anterior–posterior plane. The findings demonstrate excellent agreement between both methods and support the lateral AAL approach as a reliable alternative for liver length assessment in term and preterm neonates.

In neonatal intensive care, there are many clinical situations in which the upper abdominal ultrasound window is limited or inaccessible, making standard frontal liver measurement difficult or even impossible. These include the presence of adhesive electrodes for vital sign or transcutaneous CO₂ monitoring, which are often placed in the right upper abdomen and may obstruct the frontal AAL window. Removing or repositioning such attachments is often associated with stress and pain for the infant. This is particularly critical in preterm neonates, whose immature skin is highly susceptible to injury and subsequent infection [15, 16]. Furthermore, minimizing handling is essential in this vulnerable population, as even minor interventions can influence neurological outcomes [17, 18]. Additional limitations arise from abdominal stoma sites and large plasters or scars following surgical procedures, such as laparotomies for necrotizing enterocolitis (NEC), volvulus, or intestinal atresia [19]. Congenital abdominal wall defects such as omphalocele or gastroschisis may also obstruct the frontal ultrasound window.

The lateral approach offers several practical advantages in such scenarios. It enables liver measurements without applying pressure on the costal arch and often avoids the need to manipulate adhesive materials. Additionally, it allows simultaneous visualization of the right kidney, which facilitates comparison of echogenicity and assessment of renal morphology. The lateral scanning plane also provides a favorable ultrasound window for evaluating hepatic vessels (portal vein, hepatic artery, hepatic veins), bile ducts, and abdominal vessels such as the aorta and inferior vena cava, often with less interference from intestinal gas.

In this study, the lateral measurement method was directly compared with the established frontal AAL technique. Although the Wilcoxon test revealed a statistically significant difference between the two methods, the actual mean difference was only − 0.038 cm, which is clinically negligible. This underscores the important distinction between statistical significance and clinical relevance [20]. The high correlation (*r* = 0.9971) shows that the two methods systematically agree strongly with each other. In addition, Bland–Altman analysis demonstrated that 99.1% of all measurements fell within narrow limits of agreement, underlining the practical equivalence of the two techniques.

**Fig. 6:**
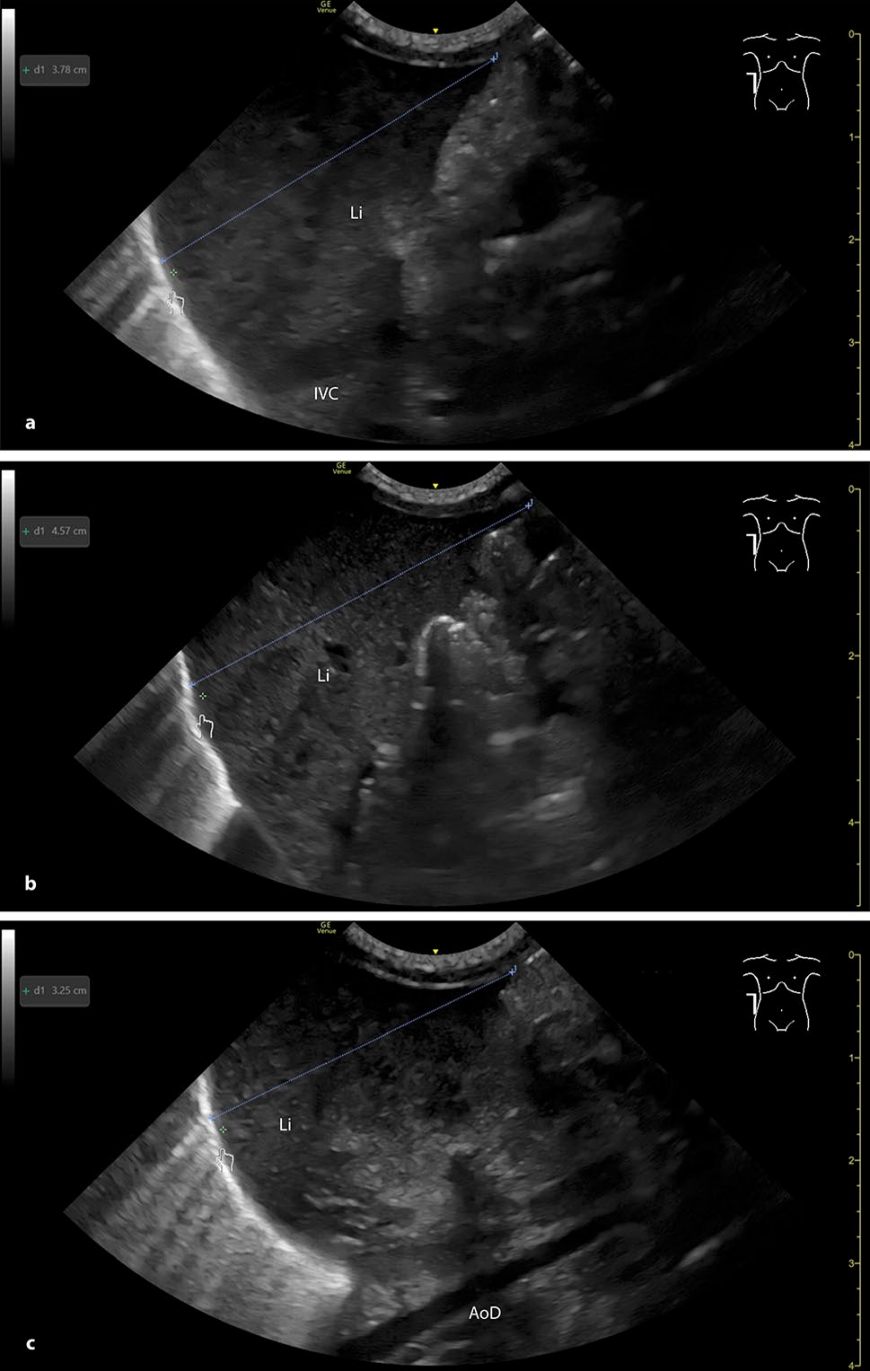
Sonographic craniocaudal liver length measurement in the right AAL in the lateral plane. **a** the probe is held parallel to the mattress (correct technique). **b** the probe is tilted cranially. **c** the probe is tilted caudally. AAL: anterior axillary line, AoD: descending aorta, IVC: inferior vena cava, Li: liver

Reproducibility was also demonstrated: a subset of patients underwent repeated measurements with a mean interval of 31.81 days, confirming that the lateral approach yields stable results over time. However, precise probe positioning is crucial for measurement accuracy. The transducer must be placed exactly in the lateral AAL and aligned parallel to the patient's mattress. Tilting the probe cranially or caudally led to inaccurate values (Fig. 6a–c), highlighting the need for a standardized technique.

To further evaluate the robustness of the lateral AAL approach, a small interobserver substudy was conducted. The results suggested that liver length measurements can be reproduced reliably between experienced examiners under standardized conditions. While the sample size was limited, these findings offer preliminary support for the applicability of the method beyond a single operator. Within both examiners, frontal and lateral measurements were internally consistent and showed no statistically significant differences. However, for Examiner 2, the p-value in the frontal–lateral comparison was close to the conventional threshold for significance (*p* = 0.0628). This may reflect minor variation related to individual technique and highlights the importance of precise and standardized probe positioning when applying the method in clinical practice.

Taken together, these findings suggest that the lateral AAL approach represents a technically robust and potentially more practical alternative to the frontal measurement—particularly in neonatal intensive care, where standard access is frequently limited and minimal handling is essential.

### Strengths and limitations of the study

One of the main strengths of this study lies in its methodological consistency: all liver measurements were performed by a single experienced examiner using the same ultrasound system, probe, and standardized technique. This approach minimized variability and ensured that any differences observed were attributable to the measurement plane itself rather than external factors. Such internal standardization is particularly important in studies comparing sonographic techniques, as it allows for a focused and reproducible evaluation of methodological differences under controlled conditions.

To complement this design, a small interobserver substudy was subsequently conducted in a separate group of neonates. Liver length measurements in both frontal and lateral AAL views were independently performed by two experienced examiners. The results indicated consistent measurements under standardized conditions, suggesting that the lateral approach may be reproducible across different examiners. However, the limited sample size warrants cautious interpretation.

Another strength lies in the practical feasibility of the lateral approach in real-life clinical settings. The study was conducted in a level III NICU and included both term and preterm infants, including extremely low birth weight infants (ELBWIs). The lateral approach proved to be technically feasible even in this challenging patient group, where small body and organ size can present significant challenges for standard abdominal imaging. In a subset of patients, repeated measurements on different days further demonstrated the reproducibility of the lateral method over time. This technical consistency provided a foundation for evaluating the method’s potential role in clinically relevant liver assessment.

Liver length is an established parameter for identifying hepatomegaly in neonates and served as a key clinical motivation for this study. However, diagnostic performance could not be formally assessed, as no systematic evaluation for hepatomegaly was possible within this cohort. Most infants did not present with suspected pathological liver enlargement, and 41 out of 107 examinations were performed in infants measuring less than 40 cm in body length. For this group, no validated normative liver size values exist, as currently available reference values for the DEGUM-recommended measurement planes begin at 40 cm body length [21]. Among the infants ≥ 40 cm, two showed liver lengths exceeding + 2 SD in the AAL, and both were reliably identified as enlarged by the lateral measurement method as well. The absence of confirmed pathological cases and of normative reference values for a substantial proportion of the cohort limits conclusions about clinical applicability in detecting hepatomegaly.

Blinding of the examiner was not feasible, as the probe position inherently revealed the measurement approach. This represents a potential source of observer bias and must be considered a methodological limitation of the study. All measurements were, however, performed by experienced examiners using a standardized protocol and consistent anatomical landmarks, which served to minimize subjectivity as far as possible under the given conditions. No comparison with imaging modalities such as MRI or 3D ultrasound was included. However, MRI is not routinely feasible in this patient population due to the need for sedation or general anesthesia, which cannot be ethically justified for research purposes alone. In contrast, 3D ultrasound may be a useful tool for future investigations, but was not available in our setting and was beyond the scope of this study. More importantly, the aim of this study was not to validate sonographic liver measurements against a radiological gold standard, but to assess whether an alternative sonographic approach yields results comparable to the established standard, within the same imaging modality.

All examinations were conducted in supine position. The lateral approach may also be applicable in prone infants—a position frequently used to improve oxygenation and feeding tolerance in neonatal care [22]. While this was not investigated in the present study, it represents a relevant potential application that warrants further exploration.

## Conclusion

In neonatal intensive care, liver length measurement via ultrasound is a well-established diagnostic tool. However, frontal access is often limited due to adhesive electrodes, stoma appliances, or postoperative coverings. This study shows that the lateral AAL measurement yields liver length values equivalent to those obtained with the standard frontal approach. Agreement between both methods was high, and the lateral technique proved to be technically feasible and consistent, even in small preterm infants. In addition, the lateral approach offers practical advantages by minimizing manipulation of sensitive attachments and reducing pressure on the costal arch—thereby supporting minimal handling, an essential aspect of neonatal care, particularly in preterm and medically fragile infants. Taken together, these findings indicate that the lateral AAL measurement may serve as a robust alternative to the standard method. However, the generalizability of our results remains limited, as all primary measurements were performed by a single examiner as part of a deliberately standardized study design. A small supplementary interobserver substudy provided initial support for reproducibility across users, but further validation in broader clinical settings is required.

In infants with body lengths ≥ 40 cm, where normative reference values for craniocaudal liver length are available, the close agreement between frontal and lateral measurements suggests that the lateral approach may be considered for clinical interpretation in selected settings. Additional studies are warranted to confirm user-independent applicability, establish reference values for liver size in smaller neonates (< 40 cm), and evaluate the diagnostic performance of the lateral method for detecting hepatomegaly. If the approach is to be applied beyond infancy, comparative validation in older pediatric populations will also be necessary.

## Supplementary Information

Below is the link to the electronic supplementary material.

Supplementary material 1 (DOCX 301 kb)

## Data Availability

The datasets generated and analyzed during this study are not publicly available due to patient confidentiality regulations but are available from the corresponding author upon reasonable request.
