# Supplementary material for: Turning the tide (and the probe)—sonographic liver measurement in the right lateral anterior axillary line is a reliable alternative to the standard frontal approach in term and preterm infants
Source: J Ultrasound. 2025 Jul 5;28(3):701–8. doi: 10.1007/s40477-025-01047-2 (PMC12496367; doi:10.1007/s40477-025-01047-2)
Supplement: Supplementary file 1 — Supplementary material 1 (DOCX 301 kb) [file 40477_2025_1047_MOESM1_ESM.docx]

**Supplemental Material:**

**Table S1** Demographic and clinical characteristics of the interobserver population

| Characteristics | Value^*^ |
| --- | --- |
| Number of infants | 17 |
| Number of examinations | 17 |
| Term or preterm | Term: 2 (12%)  Preterm: 15 (88%) |
| Sex | Female: 9 (53%)  Male: 8 (47%) |
| Gestational age (p.m., weeks) | 31 ^2^/_7_ ± 4  Range: 23 ^2^/_7_ - 38 ^3^/_7_ |
| Birth weight (kg) | 1.74 ± 0.69  Range: 0.61 - 3.08 |
| Birth length (cm) | 42.2 ± 5.39  Range: 31 - 50 |
| Age at first examination (days) | 31.1 ± 25  Range: 2 - 92 |
| Weight at first examination (kg) | 2.28 ± 0.43  Range: 1.45 - 2.87 |
| Length at first examination (cm) | 46 (45 - 47)  Range: 35 - 50 |

* = Normally distributed data are presented as mean ± SD, whereas non-normally distributed data are shown as median (IQR)

**Table S2** Comparison and interobserver agreement of frontal and lateral liver measurements

| Organ | Examiner No.1 | Examiner No. 2 | p-value | ICC | PCC |
| --- | --- | --- | --- | --- | --- |
| Liver length in frontal AAL (cm), n = 17 | **5.15** ± 0.65  Range: 3.9 - 6.4 | **5.10** ± 0.59  Range: 4.1 - 6.1 | p = 0.2078 | 0.963 | 0.9686 |
| Liver length in lateral AAL (cm), n = 17 | **5.14** ± 0.61  Range: 4 - 6.1 | **5.20** ± 0.64  Range: 4.1 - 6.2 | p = 0.2701 | 0.941 | 0.9426 |

Values are presented as mean ± standard deviation and range. p-values refer to paired t-tests, p > 0.05 indicates no significant difference between examiners. ICC: Intraclass Correlation Coefficient, PCC: Pearson Correlation Coefficient. AAL: anterior axillary line

**Table S3** Comparison and within-examiner agreement of frontal and lateral liver measurements

| Examiner | Frontal AAL (cm), n = 17 | Lateral AAL (cm), n = 17 | p-value | PCC |
| --- | --- | --- | --- | --- |
| Examiner No.1 | **5.15** ± 0.65  Range: 3.9 - 6.4 | **5.14** ± 0.61  Range: 4 - 6.1 | p = 0.7731 | 0.9679 |
| Examiner No.2 | **5.10** ± 0.59  Range: 4.1 - 6.1 | **5.20** ± 0.64  Range: 4.1 - 6.2 | p = 0.0628 | 0.9460 |

Values are presented as mean ± standard deviation and range. p-values refer to paired t-tests, p > 0.05 indicates no significant difference between measurement methods within the same examiner. PCC: Pearson Correlation Coefficient. AAL: anterior axillary line


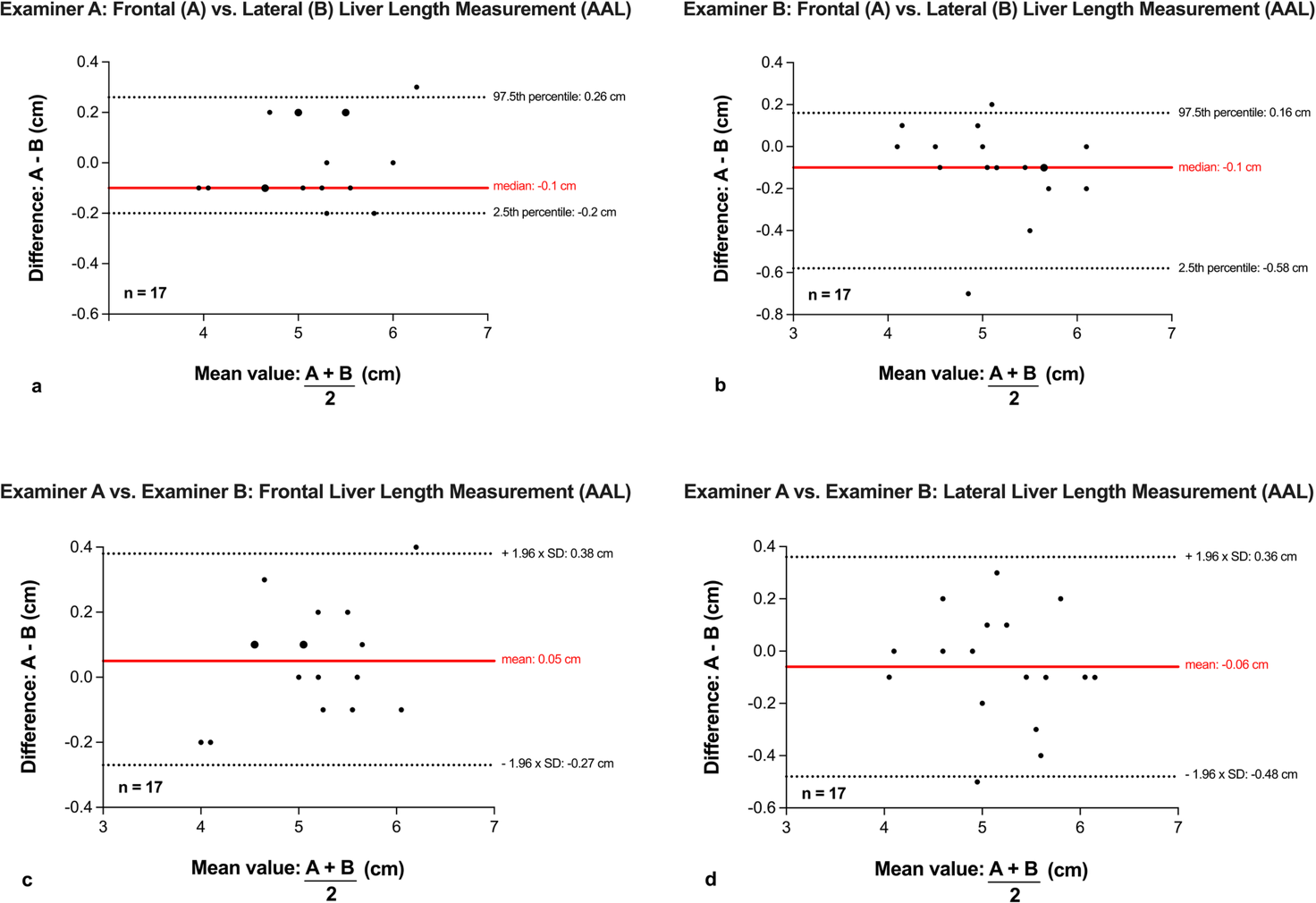
**Figure S1** Bland-Altman plots to visualize agreement between paired liver length measurements

Each point represents a measurement pair, with the x-axis showing the mean of the two values (cm) and the y-axis the difference (cm). The middle line indicates the median or mean difference, depending on data distribution, while the dashed lines represent the limits of agreement. For comparisons with non-normally distributed differences (a and b), limits were calculated using the 2.5th and 97.5th percentiles. For normally distributed differences (c and d), limits were calculated as the mean difference ± 1.96 standard deviations. Overlapping data points are shown as larger dots. The comparisons were defined as follows: a = Examiner A, frontal vs. lateral AAL; b = Examiner B, frontal vs. lateral AAL; c = Examiner A vs. Examiner B, frontal AAL; d = Examiner A vs. Examiner B, lateral AAL. AAL: anterior axillary line
